# Check Your Shopping Cart: DNA Barcoding and Mini-Barcoding for Food Authentication

**DOI:** 10.3390/foods12122392

**Published:** 2023-06-16

**Authors:** Tommaso Gorini, Valerio Mezzasalma, Marta Deligia, Fabrizio De Mattia, Luca Campone, Massimo Labra, Jessica Frigerio

**Affiliations:** 1 FEM2-Ambiente, Piazza della Scienza 2, 20126 Milano, Italy; tommaso.gorini@fem2ambiente.com (T.G.); valerio.mezzasalma@fem2ambiente.com (V.M.); fabrizio.demattia@fem2ambiente.com (F.D.M.); 2 Department of Scienze Agrarie, Forestali e Alimentari, University of Turin, Via Verdi 8, 10124 Torino, Italy; marta.deligia@tiscali.it; 3 Department of Biotechnology and Biosciences, University of Milano-Bicocca, Piazza della Scienza 2, 20126 Milano, Italy; luca.campone@unimib.it (L.C.); massimo.labra@unimib.it (M.L.)

**Keywords:** DNA barcoding, food fraud, species identification, food quality, food safety, food supply chain

## Abstract

The molecular approach of DNA barcoding for the characterization and traceability of food products has come into common use in many European countries. However, it is important to address and solve technical and scientific issues such as the efficiency of the barcode sequences and DNA extraction methods to be able to analyze all the products that the food sector offers. The goal of this study is to collect the most defrauded and common food products and identify better workflows for species identification. A total of 212 specimens were collected in collaboration with 38 companies belonging to 5 different fields: seafood, botanicals, agrifood, spices, and probiotics. For all the typologies of specimens, the most suitable workflow was defined, and three species-specific primer pairs for fish were also designed. Results showed that 21.2% of the analyzed products were defrauded. A total of 88.2% of specimens were correctly identified by DNA barcoding analysis. Botanicals (28.8%) have the highest number of non-conformances, followed by spices (28.5%), agrifood (23.5%), seafood (11.4%), and probiotics (7.7%). DNA barcoding and mini-barcoding are confirmed as fast and reliable methods for ensuring quality and safety in the food field.

## 1. Introduction

The complexity of the food supply network, including disruption due to COVID-19 and climate change, can make food products more vulnerable to fraud and substitution. It is difficult to quantify the impact of fraud on the whole food field because not all fraud is detected. However, food safety experts interviewed by Spielman estimated the impact of fraud on the food industry to be in excess of USD 50 billion annually [1]. Food fraud can occur anywhere in the food supply chain, from the seed supply to food packaging. Mislabelling (20.7%), artificial enhancement (17.2%), and substitution (16.4%) were the most commonly reported types of fraud [2]. Mislabelling has been frequently reported in the literature: up to 57% in processed meat products [3,4], up to 80% in fish filets [5,6], and up to 80% in dairy products [7]. Concerning the herbal supplements field, a global survey showed that 27% of herbal products commercialized in the global marketplace are adulterated. The most defrauded regions are Australia (79% mislabelled products) followed by South America (67% mislabelled products) [8]. Undeclared species substitution in food products might also represent an important health threat to allergic consumers because of the introduction of food allergens, such as different kinds of nuts and mollusks [9] or poisonous plants [10]. Even though it is a current problem, agribusiness has not paid sufficient attention to this issue. Most fraud is harmless, and this leads to a lack of attention. Nevertheless, the consumers have a large interest in the quality of food. McCallum and colleagues investigate consumers’ willingness to pay for premium products to reduce risk and uncertainty related to food fraud, showing that consumers are willing to pay for premium products to avoid food fraud and purchase an authentic product [11]. In this regard, blockchain has emerged as a promising technology that allows users to trace food products and eliminate or reduce harmful food fraud.

Treiblmaier and Garaus investigate how the use of blockchain to trace food products impacts consumers’ perception of product quality, finding that blockchain labels help to strengthen consumers’ perceived quality of food products which, in turn, increases their purchase intention [12]. Introducing DNA analysis into supply chain control could increase consumers’ confidence and consequently the budget allocated for food shopping. DNA barcoding has been frequently used in the literature for food authentication and supply chain control [13,14,15].

The application of DNA barcoding in food authentication is rooted in the concept of using short and standardized DNA sequences to differentiate between species. The technique targets specific regions of the genome, such as the mitochondrial DNA (mtDNA) or chloroplast DNA (cpDNA), which exhibit sufficient variability among species while maintaining conserved regions within the same species [16,17]. By comparing the barcode sequences obtained from unknown samples with well-curated reference databases, such as ncbi (https://www.ncbi.nlm.nih.gov/nucleotide/ accessed on 1 May 2023) and BOLD (https://www.boldsystems.org/ accessed on 1 May 2023), DNA barcoding allows for the identification of species present in food products, thereby enabling the detection of fraudulent practices.

One of the key advantages of DNA barcoding is its ability to detect adulteration and substitution in complex food matrices [18]. The technique can differentiate between closely related species or detect the presence of non-declared ingredients, even in processed or highly fragmented products. For instance, in cases where premium and expensive seafood species are substituted with cheaper alternatives, DNA barcoding can expose such fraudulent activities by identifying the true species present in the sample [19]. Similarly, it can detect the presence of allergenics that may pose health risks to consumers. Furthermore, DNA barcoding can aid in the identification of geographical origins or specific cultivars, providing valuable information regarding product quality, cultural heritage, and compliance with geographical indication regulations [20].

The use of DNA barcoding in combating food fraud has gained significant attention worldwide. Governments, regulatory agencies, and industry stakeholders recognize its potential to ensure food authenticity, protect consumer rights, and maintain market integrity. In recent years, various countries and international organizations have established initiatives and regulations to promote the adoption of DNA barcoding as a standard practice in food authentication. These include The EU Agri-Food Fraud Network (FFN), the United States Food and Drug Administration’s (FDA) GenomeTrakr program, and the International Organization for Standardization’s (ISO) guidelines on DNA-based methods for food authenticity testing.

Despite its numerous benefits, DNA barcoding is not without limitations. Challenges related to sample preparation, DNA extraction, database completeness, and the availability of suitable reference materials need to be addressed for wider adoption and successful implementation. Furthermore, ongoing advancements in DNA sequencing technologies, bioinformatics tools, and reference databases are vital to enhance the accuracy, efficiency, and reliability of DNA barcoding in food fraud detection.

This study aimed to identify several workflows of DNA barcoding for supply chain control in different food fields. A total of 38 companies, operating in 5 different fields (seafood, botanicals, agrifood, spices, and probiotics) supplied some of their high-selling products for a total of 212 specimens. Among these samples we can find fish filets, herbal teas, truffles, caviar, canned fish, processed products, powders, plant extracts, food supplements, flours, etc., a mix of products that can be considered representative of a supermarket shopping cart. The technical goals of the study are to (i) define the most suitable extraction methods for food matrices, (ii) identify the most suitable barcode region useful for different types of products (i.e., fresh, processed, etc.) and designed primer pairs when necessary, and (iii) estimate the ability of DNA barcoding tools to assess fraud in high-selling products.

## 2. Materials and Methods

### 2.1. Specimen Collection

The specimen collection was based on some defined criteria: (i) the most counterfeit species according to the literature were collected, (ii) sampling of the same species belonging to different companies was preferred, and (iii) when possible, raw/fresh, intermediate, and final products were collected. A total of 46 companies operating in the food field were contacted to join this study. The companies were chosen considering the food field operating (seafood, agrifood, spices, botanicals, and probiotic) with the aim to cover the most defrauded fields. A total of 38 companies agreed to participate in the project and a total of 212 specimens were collected (Table A1). In this study, we analyzed different typologies of products, from fresh (fish filets) to highly processed products (food supplements).

### 2.2. DNA Extraction

Considering the wide typology of specimens tested in this study, different commercial kits and extraction methods were chosen based on the literature [21,22,23,24]. For seafood specimens (fresh and intermediate specimens), Tissue Genomic DNA Extraction (Fisher Molecular Biology, Rome, Italy) (TGF) was selected and for more difficult products, such as canned fish and products preserved in oil and brine; the ReliaPrep™ gDNA Tissue MiniPrep System (Promega, Milan, Italy) (RPP) was tested/used with a modification to the protocol. The products preserved in brine were washed three times with a physiological solution (NaCl 0.7%), mixing overnight at room temperature. Canned specimens were pretreated in order to clean the tissue from the conservation liquid, such as oil (vegetable and olive); briefly, oil and lipids were removed by soaking in chloroform/methanol/water (1:2:0.8) and mixing overnight at room temperature [24]. 

For agrifood products, spices, and botanicals, a DNeasy Plant Kit (QIAGEN, Milan, Italy) (DPQ) was used following the instructions. For more complex samples belonging to these fields, such as phytoextract, the CTAB method was also applied [25]. The CTAB method allows us to start from a higher amount of material (1 g) and to harvest all the DNA in the solution.

Finally, for probiotic specimens, QIAamp DNA Microbiome Kit (QIAGEN) (QAQ) was used. In Table A1 are shown all the extraction methods used for all specimens. Purified gDNA was checked for concentration and purity by using a Qubit 2 Fluorometer and Qubit dsDNA HS Assay Kit (Invitrogen, Carlsbad, CA, USA).

### 2.3. Barcode Region Selection

A universal set of DNA barcoding markers for each product was tested. Specifically, different primer pairs were selected for animals, plants, fungi, and bacteria.

#### 2.3.1. Animal DNA Barcoding

The amplification efficiency of the barcode region is associated with the primer pairs. A primer pair specific for a universal barcode region should be versatile across a wide range of animal species and have high affinity to DNA templates. Nevertheless, sometimes the universal primers are not applicable for certain taxa or specimens and it is necessary to redesign primers, as for some specimens in this study [26]. The barcode regions chosen for animal identifications were the mitochondrial markers COI (Cytochrome c oxidase I), RNA 16S (16S ribosomal RNA), CytB (Cytochrome b), and Control Region (DLoop). For the species *Dicentrarchus labrax*, *Katsuwonus pelamis*, *Thunnus* sp., primer pairs for DNA barcoding and mini-barcoding, respectively, were designed in silico in this study. For *Dicentrarchus labrax,* the COI region was identified as the most suitable for species identification, while for *Katsuwonus pelamis* and *Thunnus* spp., the control region (CR) was chosen based on the literature [22]. All nucleotide sequences of the COI gene and control region (CR) were obtained from NCBI Nucleotide for *Dicentrarchus* spp., *Katsuwonus pelamis*, and *Thunnus* spp., respectively, and were aligned using ClustalW2 software (www.ebi.ac.uk/Tools/msa/clustalw2/ accessed on 1 May 2023). The most conserved regions for *Dicentrarchus* sp., *Katsuwonus pelamis,* and *Thunnus* spp. were identified using Bioedit software and primer pairs specific for the genus *Dicentrarchus* spp. and *Thunnus* spp. and the species *Katsuwonus pelamis* were de novo designed. Primer pairs were tested with Primer–Blast tool available from NCBI (www.ncbi.nlm.nih.gov/tools/primer-blast/ accessed on 1 May 2023) to verify the specificity. Primer sequences are shown in Table 1.

#### 2.3.2. Plant DNA Barcoding

Starting from 2005, mitochondrial, plastid, and nuclear genomes were studied to identify a barcode universal region for plants [39,40,41,42] and four gene regions (*rbcL*, *matK*, *trnH-psbA*, and ITS) have been chosen as the standard DNA barcodes in most applications for plants [43,44,45]. In this study, all of these barcode regions were tested. However, recently, some manuscripts described the efficacy of mini-barcode regions (i.e., the analysis of smaller genome portions—100–150 bp—usually associated with the largest DNA barcodes) for the identification of processed plant extracts [46,47]. Furthermore, in this study, a DNA mini-barcoding barcode (*rbcL* mini-barcoding) was tested for plant extracts. Primer sequences are shown in Table 1. The different plant regions chosen for each species were defined after an in silico analysis; the sequences for the DNA barcoding marker chosen in this study were downloaded from NCBI Nucleotide database (https://www.ncbi.nlm.nih.gov/nucleotide/ accessed on 1 May 2023). Sequences were aligned using the online tool Muscle (https://www.ebi.ac.uk/Tools/msa/muscle/ accessed on 1 May 2023) and manually edited using Bioedit. Haplotypes were collapsed by using the online tool Fabox (https://users-birc.au.dk/palle/php/fabox/ accessed on 1 May 2023). Finally, each haplotype was compared to the online database using the BLAST algorithm (https://blast.ncbi.nlm.nih.gov/Blast.cgi accessed on 1 May 2023). The best performing plant markers in terms of identification were chosen and selected for the analysis.

#### 2.3.3. Fungi DNA Barcoding

The most common barcode region for fungi identification is ITS [48,49,50]. El Karkouri and colleagues also tested this region for truffles, finding the efficiency for species identification for *Tuber* spp. Genera [51]. In this study, the ITS barcode region was also chosen for DNA barcoding analysis. Primer sequences are shown in Table 1.

#### 2.3.4. Bacteria DNA Barcoding

For bacteria identification, the 16S rRNA gene is used. It is a common housekeeping gene in all prokaryotic organisms. This gene is the most used in bacterial study because (i) it is present in almost all bacteria, (ii) the function of the 16S rRNA gene over time has not changed, suggesting that random sequence changes are a more accurate measure of the evolution, and (iii) the 16S rRNA gene (1500 bp) is large enough for informatics purposes, even if, for DNA barcoding, a smaller region is analyzed [52]. Primer sequences are shown in Table 1.

### 2.4. DNA Amplification and Identification

A standard PCR amplification was performed using PCR Mix Plus (A&A Biotechnology, Danzica, Poland) following the manufacturer’s instructions in a 25 μL reaction containing 1 μL 10 mM of each primer and 3 μL of gDNA (about 20–50 ng). PCR cycles differ in relation to the primer pairs used. All the PCR programs are shown Appendix A Table A2. The amplicon was visualized by electrophoresis on agarose gel using 1.5% agarose Tris-acetate-EDTA (TAE) gel. Purified amplicons were bidirectionally sequenced by Sanger at Eurofins Genomics (Ebersberg, Germany). After manual editing, primer removal, and pairwise alignment, all the tested samples’ (Table A2) identities were assessed by adopting a standard comparison approach against the GenBank database with BLASTn [53]. Each barcode sequence was taxonomically assigned to the species with the nearest matches (maximum identity > 99% and query coverage of 100%).

## 3. Results and Discussion

DNA extraction was successful for 187 specimens out of 212, with high DNA quality and good yield (i.e., 3.2–27.4 ng/μL). The presences in the public databases of the sequences for all the species considered in our study were checked and confirmed. For 25 specimens (11,8% of total), 22 for botanicals and 3 for spices, the extracted DNA was not suitable for the analysis in terms of quantity and quality (Table A1). Concerning the identification, for most of the samples (88.2%), it was possible to identify the species, proving the suitability of the barcode region selected. A total of 45 samples out of 212 were defrauded for a total of 21.2% of detected fraud (Table A1).

Considering the results of this study, the most defrauded products were botanicals, with 28.8% of substitution or contamination (Table 2). To identify the contaminants, further analysis, such as Next Generation Sequencing (NGS), is necessary [54].

**Figure 1:**
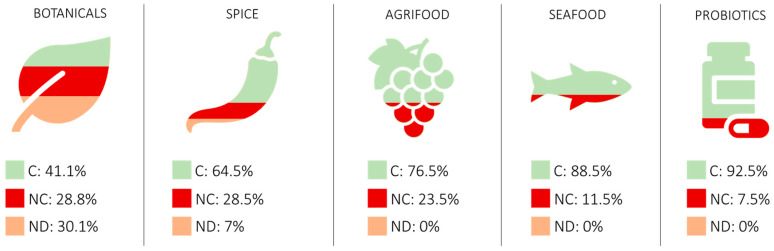
Infographics represent the percentage of compliant, non-compliant, and specimens with no DNA detected (C, NC and ND) for all the field analyzed.

Almost all specimens were impossible to identify by morphological methodology, because they were treated, in the form of powder or capsule. This value is in line with the percentage presented by Ichim and colleagues, who showed that 27% of the herbal products commercialized in the global marketplace are adulterated [8]. In the same way, the higher percentage of specimens without detectable DNA (31.4%) were botanicals too (Figure 1, Table 2). This value can be explained considering that more than half of the specimens (51 of 70) undergo industrial pre-treatment, such as high temperatures, use of solvent (ethanol, glycerol), and other industrial treatments such as CO_2_ supercritical extraction. These industrial processing steps degrade, fragment, and precipitate DNA. In a previous study of ours [46], we evaluated the capability of DNA barcoding identification for botanicals (phytoextract and botanicals). We found that phytoextracts obtained through hydroalcoholic treatment, with the lower percentage of ethanol (<40%) and aqueous processing at low temperature, had a major rate of sequencing and identification success. In this study, we obtained similar results, with a success of identification for liquid aqueous phytoextracts with a low percentage of ethanol (<40%) (i.e., DIF_74, DIF_75, DIF_138, etc.) and an incapability to detect DNA in the other typology of specimens. 

After botanicals, the spice sector revealed 28.5% of defrauded products. Our results are in line with the study of Cottenet and colleagues [55]. In most of the non-compliant samples (10 of 12), we did not find a substitution, but a contamination. In some cases (DIF_147 and DIF_173), we were able to identify the genera; in all the remaining samples we obtained multiple sequences and it was impossible to identify any species or genera. This means that the contamination in those samples is high and it is possible that multiple species coexist, as indicated in the literature [56].

Although in the agrifood samples the fraud percentage is lower than botanicals and spices, we face a substitution case of fraud for all the cases. Sample DIF_193 was declared *Tuber brumale* but was identified as *Tuber melanosporum.* These two species, although similar, can be distinguished by morphological analysis. Analyzing the gleba, the *Tuber melanosporum*, known as the black truffle or Périgord truffle, it is very dark, tending to purplish-black and with fine white veins, while the *Tuber brumale*, commonly known as winter truffle or musky truffle, is grey-brownish with large and sparse veins. The interesting fact is that *Tuber melanosporum* is more expensive than *Tuber brumale.* In this case we are facing an involuntary substitution that damages the company but not the consumers. For this reason, the control of the supply chain is important, not only to offer a high-quality product, but also to avoid mistakes that can damage the company itself. The frauds detected in seafood products are not in line with the literature, which declares a percentage of 25–30% of mislabelling [57], while we detected a lower percentage (11.4%). This data can be explained considering that the specimens analyzed were collected directly from the company, assuming that all the samples were compliant. In all cases we faced a case of mislabelling, which is a false claim or distortion of the information provided on the label/packaging. The specimens DIF_009, DIF_069, and DIF_070 were different species of the same genus. They were probably an unintentional fraud. Nevertheless, the specimens DIF_008, DIF_027, DIF_028, DIF_041, and DIF_048 were found to be a totally different genus. The most serious case is the sample DIF_041. This specimen was a processed product and the species was impossible to detect by morphological analysis. It was declared as *Theragra chalcogramma* but was found to be *Lepidopsetta polyxystra. Theragra chalcogramma* belongs to the order Gadiformes and is commonly called “Alaska pollock”, while *Lepidopsetta polyxystra* belongs to the order Pleuronectiformes and is a flat fish commonly called “Northern rock sole”. The criticality of the seafood sector is that companies buy filets or semi-processed fish, unlike other sectors where the starting material is already ground or processed (e.g., botanicals, spices, etc.). This highlights a problem in the control of the supply chain. Finally, the probiotics sector was found to have the lowest percentage of fraud (7.7%). Moreover, we found that the specimen DIF_210, declared *Bifidobacterium bifidum*, was contaminated with other bacteria. There was probably an unintentional contamination in the production site with another probiotic. Recent studies have demonstrated that probiotic contamination with other probiotics is a common occurrence. For example, a study by Lewis and colleagues found that the contents of many bifidobacterial probiotic products analyzed in their study differ from the ingredient list, sometimes at a subspecies level. Only 1 of the 16 probiotics perfectly matched its bifidobacterial label claims in all samples tested [58]. The implications of probiotic contamination can vary depending on the specific strains involved and the intended use of the probiotic product. In some cases, the presence of unintended probiotics may be harmless or even beneficial. However, there is also a risk of introducing harmful or pathogenic microorganisms that may compromise the safety and efficacy of the probiotic product.

Considering the data from Table 2, it is possible to notice how processed products (such as botanicals, agrifood, and spices) have a significantly higher percentage of fraud. This is because the product, being crushed, transformed, or otherwise not in its whole form, is more difficult to identify morphologically and therefore fraud is more easily carried out.

To conclude, the extraction methods retrieved from the literature and tested in this study seem to be suitable for the chosen products, due to the DNA extraction success of 187 specimens out of 212. Moreover, most of the samples were identified at the species level, so in this study the most suitable barcode regions useful for different types of products were identified. The DNA barcoding approach, given its maturity and its wide application in the last twenty years, could be used in strategic points of the food supply chain: customs, goods management office, but also directly in medium-large companies and in the GDO. Nowadays some companies use DNA analysis to check their suppliers and to ensure customers a quality product, but this technology, although widely used in the scientific environment, is not yet fully accepted by the final consumer. Raising awareness and citizen science will be needed to convey the importance and potential of this approach. In conclusion, this study contributes to the growing body of research on DNA barcoding for species identification in the food industry.

## 4. Conclusions

Given the results of this study, DNA analysis provides a powerful tool for detecting and identifying contaminants in commercial food products, enabling manufacturers and regulatory authorities to take appropriate action to ensure the quality and safety of these products. The results confirm the suitability and reliability of DNA barcoding and mini-barcoding as fast and effective methods for ensuring quality and safety in the food field. Moreover, techniques such as LAMP, RPA, BAR-RPA, Bar-HRM, and minION have made DNA-based methods more affordable, as they require cheaper instruments and protocols [22,59,60]. However, some challenges, in particular in relation to non-conformances observed in botanicals and spices, remain an issue to investigate. Nevertheless, by addressing these technical and scientific issues and implementing standardized workflows, DNA barcoding can play a crucial role in combating food fraud and enhancing traceability in the food supply chain, thus ensuring consumer confidence and facilitating regulatory compliance.

## Figures and Tables

**Table 1:** List of primer name, gene target, primer sequence (5′->3′), bp of the fragment obtained, annealing temperature, taxonomic target, and reference.

Primer Name	Gene	Primer Sequence (5′->3′)	bp	Ta °C	Target	Reference
Cox1_Ward_FishF1	COI	F: TCAACCAACCACAAAGACATTGGCAC	655	55 °C	Bony fish	[27]
Cox1_Ward_FishR1		R: TAGACTTCTGGGTGGCCAAAGAATCA				
Cox1_Ward_FishF2	COI	F: TCGACTAATCATAAAGATATCGGCAC	616	55 °C	Bony fish	[27]
Cox1_Ward_FishR2		R: ACTTCAGGGTGACCGAAGAATCAGAA				
LCO 1490	COI	F: GGTCAACAAATCATAAAGATATTGG	700	47 °C	Crustaceans and cephalopods	[28]
HCO 2198		R: TAAACTTCAGGGTGACCAAAAAATCA				
16sar-L	16S rRNA	F: CGCCTGTTTAYCAAAAACAT	571	57 °C	Animal universal	[29]
16sbr_H		R: CCGGTCTGAACTCAGATCACGT				
GLUDG	Cytb	F: TGACTTGAARAACCAYCGTTG	1140	52 °C	Animal universal	[30]
C61221H		R: CTCCAGTCTTCGRCTTACAAG				
Tuna_CR_F	CR	F: GCAYGTACATATATGTAAYTACACC	236	58 °C	*Thunnus* spp.	[31]
Tuna_CR_R		R: CTGGATGGTAGGYTCTTACTGCG				
Tuna_CR_F	CR	F: GCAYGTACATATATGTAAYTACACC	80	52 °C	*Thunnus* spp.	[31]/This study
Tuna_minibar_R2		R: GAYATATGAATAKTTWSRTAC				
Sco5S_F	ITS	F: CTCACTGTTACAGCCTG	120	48 °C	*Scomber* spp.	[19]
Sco5S_R		R: CAAACACATGCTATCCTT				
Katw_F	CR	F: GCGAGATYTAAGACCTACCACG	80	54 °C	*Katswonus* spp.	This study
Katw_R		R: GAGCTGGTTGGTCTCTT				
Dlab_F	COI	F: TCTTATTCTCCCCGGGTTCG	186	59 °C	*Dicentrarchus* spp.	This study
Dlab_R		R: GATGTGAAGTATGCGCGTGT				
rbcL_1F	rbcL	F: ATGTCACCACAAACAGAAAC	743	50 °C	Plants universal	[31,32]
rbcL724R		R: TCGCATGTACCTGCAGTAGC				
rbcL 1	rbcL	F: TTGGCAGCATTYCGAGTAACTCC	226	50 °C	Plants universal	[33]
rbcL B		R: AACCYTCTTCAAAAAGGTC				
matK_3F_KIM	matK	F: CGTACAGTACTTTTGTGTTTACGAG	636	53 °C	Plants universal	[34]
matK_1R_KIM		R: ACCCAGTCCATCTGGAAATCTTGGTT				
psbA	psbA-trnH	F: GTTATGCATGAACGTAATGCTC	300–600	53 °C	Plants universal	[35]
trnH		R: CGCGCATGGTGGATTCACAATCC				
ITS-p5	ITS	F: CCTTATCAYTTAGAGGAAGGAG	300–750	55 °C	Plants universal	[36]
ITS-u4		R: RGTTTCTTTTCCTCCGCTTA				
ITS3_KYO2	ITS	F: GATGAAGAACGYAGYRAA	300–500	55 °C	Fungi	[37]
ITS-4		R: RGTTTCTTTTCCTCCGCTTA				
P0	16S rRNA	F: GAGAGTTTGATCCTGGCTCAG	1540	54 °C	Bacteria	[38]
P6		R: CTACGGCTACCTTGTTACGA				

**Table 2:** In the table are indicated the number of specimens analyzed divided into compliant, non-compliant, and samples where DNA was not detected, also expressed in percentage.

Specimens’ Typology	Collected Specimens	Sector	Compliant (Percentage)	Non-Compliant (Percentage)	No DNA Detected (Percentage)
Fresh/raw	6	Seafood	6 (100%)	/	/
Intermediate	4		4 (100%)	/	/
Processed	60		52 (86.6%)	8 (13.3%)	/
Total	70		62 (88.5%)	8 (11.5%)	/
Fresh/raw	19	Botanicals	14 (73.7%)	5 (26.3%)	/
Intermediate	3		3 (100%)	/	/
Processed	51		13 (25.5%)	16 (31.3%)	22 (43.2%)
Total	73		30 (41.1%)	21 (28.8%)	22 (30.1%)
Fresh/raw	13	Agrifood	12 (92.3%)	1 (7.7%)	0
Intermediate	/		/	/	0
Processed	4		1 (25%)	3 (75%)	0
Total	17		14 (76.5%)	3 (23.5%)	/
Fresh/raw	18	Spice	13 (72.2%)	4 (22.2%)	1 (5.6%)
Intermediate	/		/	/	/
Processed	24		14 (58.3%)	8 (33.3%)	2 (8.3%)
Total	42		27 (64.5%)	12 (28.5%)	3 (7%)
Fresh/raw	/	Probiotics	/	/	/
Intermediate	/		/	/	/
Processed	13		12 (92.5%)	1 (7.5%)	/
Total	13		12 (92.5%)	1 (7.5%)	/

## Data Availability

The data presented in this study are available on request from the corresponding author.

## Appendix A

**Table A1:** In the table are indicated specimen code, the company, the sample typology, the declared and detected species, the field, the extraction typology, and the barcode region chosen.

Specimen Code	Company	Sample Typology	Declared Species	Detected Species	Sector	Extraction	Barcode
DIF_001	Company 1	Canned olive oil	*Thunnus albacares*	*Thunnus albacares*	Seafood	RPP	Control Region
DIF_002	Company 1	Canned olive oil	*Katsuwonis pelamis*	*Katsuwonus pelamis*	Seafood	RPP	Control Region
DIF_003	Company 1	Canned seed oil	*Thunnus albacares*	*Thunnus albacares*	Seafood	RPP	Control Region
DIF_004	Company 1	Canned olive oil	*Thunnus albacares*	*Thunnus albacares*	Seafood	RPP	Control Region
DIF_005	Company 1	Canned olive oil	*Thunnus albacares*	*Thunnus albacares*	Seafood	RPP	Control Region
DIF_006	Company 1	Brine	*Thunnus albacares*	*Thunnus albacares*	Seafood	RPP	Control Region
DIF_007	Company 1	Canned olive oil	*Scomber colias*	*Scomber colias*	Seafood	RPP	ITS
DIF_008	Company 1	Brine	*Katsuwonis pelamis*	*Thunnus albacares*	Seafood	RPP	Control Region
DIF_009	Company 1	Canned olive oil	*Thunnus obesus*	*Thunnus albacares*	Seafood	RPP	Control Region
DIF_010	Company 1	Canned olive oil	*Engraulis engrasicolus*	*Engraulis engrasicolus*	Seafood	TGF	COI
DIF_011	Company 2	Fillet	*Parapenaeus longirostris*	*Parapenaeus longirostris*	Seafood	TGF	COI
DIF_012	Company 2	Fillet	*Litopenaeus vannamei*	*Litopenaeus vannamei*	Seafood	TGF	COI
DIF_013	Company 2	Fillet	*Lates niloticus*	*Lates niloticus*	Seafood	TGF	COI
DIF_014	Company 2	Burger	*Xiphias gladius*	*Xiphias gladius*	Seafood	TGF	COI
DIF_015	Company 2	Burger	*Xiphias gladius*	*Xiphias gladius*	Seafood	TGF	COI
DIF_016	Company 2	Burger	*Oncorhynchus mykiss*	*Oncorhynchus mykiss*	Seafood	TGF	COI
DIF_017	Company 2	Burger	*Salmo salar*	*Salmo salar*	Seafood	TGF	COI
DIF_018	Company 2	Burger	*Thunnus albacares*	*Thunnus albacares*	Seafood	TGF	Control Region
DIF_019	Company 2	Burger	*Oncorhynchus mykiss*	*Oncorhynchus mykiss*	Seafood	TGF	COI
DIF_020	Company 2	Burger	*Oncorhynchus mykiss*	*Oncorhynchus mykiss*	Seafood	TGF	COI
DIF_021	Company 2	Processed product	*Oncorhynchus mykiss*	*Oncorhynchus mykiss*	Seafood	TGF	COI
DIF_022	Company 3	Fillet	*Sepia officinalis*	*Sepia officinalis*	Seafood	TGF	COI
DIF_023	Company 3	Fillet	*Nephrops norvegicus*	*Nephrops norvegicus*	Seafood	TGF	COI
DIF_024	Company 3	Fillet	*Parapenaeus longirostris*	*Parapaeneus longirostris*	Seafood	TGF	COI
DIF_025	Company 3	Fillet	*Aristeomorpha foliacea*	*Aristeomorpha foliacea*	Seafood	TGF	COI
DIF_026	Company 3	Fillet	*Loligo vulgaris*	*Loligo vulgaris*	Seafood	TGF	COI
DIF_027	Company 3	Fillet	*Todarodes sagitattus*	*Todaropsis eblanae*	Seafood	TGF	COI
DIF_028	Company 3	Fillet	*Trigloporus lastoviza*	*Chelidonichthys cuculus*	Seafood	TGF	COI/16S rRNA
DIF_029	Company 3	Fillet	*Merluccius merluccius*	*Merluccius merluccius*	Seafood	TGF	COI
DIF_030	Company 3	Fillet	*Octopus vulgaris*	*Octopus vulgaris*	Seafood	TGF	COI
DIF_031	Company 4	Processed product	*Dicentrarchus labrax*	*Dicentrarchus labrax*	Seafood	TGF	CytB
DIF_032	Company 4	Processed product	*Merluccius gayi*	*Merluccius gayi*	Seafood	TGF	COI
DIF_033	Company 4	Processed product	*Salmo salar*	*Salmo salar*	Seafood	TGF	COI
DIF_034	Company 4	Processed product	*Thunnus albacares*	*Thunnus albacares*	Seafood	TGF	Control Region
DIF_035	Company 4	Intermediate product	*Thunnus albacares*	*Thunnus albacares*	Seafood	TGF	Control Region
DIF_036	Company 4	Processed product	*Thunnus albacares*	*Thunnus albacares*	Seafood	TGF	Control Region
DIF_037	Company 4	Fillet	*Thunnus albacares*	*Thunnus albacares*	Seafood	TGF	Control Region
DIF_038	Company 5	Fillet	*Pleuronectes platessa*	*Pleuronectes platessa*	Seafood	TGF	COI
DIF_039	Company 5	Brine	*Octopus vulgaris*	*Octoupus vulgaris*	Seafood	TGF	COI
DIF_040	Company 5	Brine	*Dosidicus gigas*	*Dosidicus gigas*	Seafood	TGF	COI
DIF_041	Company 5	Processed product	*Theragra chalcogramma*	*Lepidopsetta polyxystra*	Seafood	TGF	COI/16S rRNA
DIF_042	Company 5	Brine	*Sepia officinalis*	*Sepia officinalis*	Seafood	TGF	COI
DIF_043	Company 5	Brine	*Litopenaeus vannamei*	*Litopenaeus vannamei*	Seafood	TGF	COI
DIF_044	Company 5	Brine	*Dosidicus gigas*	*Dosidicus gigas*	Seafood	TGF	COI
DIF_045	Company 5	Brine	*Thunnus albacares*	*Thunnus albacares*	Seafood	TGF	Control Region
DIF_046	Company 5	Brine	*Salmo salar*	*Salmo salar*	Seafood	TGF	COI
DIF_047	Company 5	Brine	*Gadus morhua*	*Gadus morhua*	Seafood	TGF	COI
DIF_048	Company 5	Brine	*Molva* spp.	*Brosme brosme*	Seafood	TGF	COI
DIF_049	Company 5	Processed product	*Merluccius paradoxus*	*Merluccius paradoxus*	Seafood	TGF	COI
DIF_050	Company 7	Fresh product	*Acipenser transmontanus*	*Acipenser transmontanus*	Seafood	TGF	CytB
DIF_051	Company 7	Fresh product	*Acipenser gueldenstaedtii*	*Acipenser gueldenstaedtii*	Seafood	TGF	CytB
DIF_052	Company 7	Fresh product	*Huso huso*	*Huso huso*	Seafood	TGF	CytB
DIF_053	Company 7	Fresh product	*Acipenser naccarii*	*Acipenser naccarii*	Seafood	TGF	CytB
DIF_054	Company 7	Fresh product	*Acipenser baerii*	*Acipenser baerii/ Acipenser gueldenstaedtii*	Seafood	TGF	CytB
DIF_055	Company 7	Fresh product	*Acipenser stellatus*	*Acipenser stellatus*	Seafood	TGF	CytB
DIF_056	Company 8	Intermediate product	*Thunnus* spp.	*Thunnus albacares*	Seafood	TGF	Control Region
DIF_057	Company 8	Canned olive oil	*Thunnus* spp.	*Thunnus albacares*	Seafood	RPP	Control Region
DIF_058	Company 8	Intermediate product	*Thunnus* spp.	*Thunnus albacares*	Seafood	TGF	Control Region
DIF_059	Company 8	Canned olive oil	*Thunnus* spp.	*Thunnus albacares*	Seafood	RPP	Control Region
DIF_060	Company 8	Intermediate product	*Thunnus* spp.	*Thunnus albacares*	Seafood	TGF	Control Region
DIF_061	Company 8	Canned olive oil	*Thunnus* spp.	*Thunnus albacares*	Seafood	RPP	Control Region
DIF_062	Company 8	Canned olive oil	*Engraulis encrasicolus*	*Engraulis encrasicolus*	Seafood	RPP	COI
DIF_063	Company 9	Fillet	*Dentex angolensis Poll & Maul*	*Dentex angolensis Poll & Maul*	Seafood	TGF	COI
DIF_064	Company 9	Fillet	*Synaptura* spp.	*Synaptura lusitanica*	Seafood	TGF	COI
DIF_065	Company 9	Fillet	*Psettodes* spp.	*Psettodes bennettii*	Seafood	TGF	COI
DIF_066	Company 9	Fillet	*Gadus morhua* L.	*Gadus morhua* L.	Seafood	TGF	COI
DIF_067	Company 9	Fillet	*Epinephelus costae Steindachner*	*Epinephelus costae Steindachner*	Seafood	TGF	COI
DIF_068	Company 9	Fillet	*Dosidicus gigas d’Orbigny*	*Dosidicus gigas d’Orbigny*	Seafood	TGF	COI
DIF_069	Company 9	Processed product	*Merluccius capensis Castelnau*	*Merluccius paradoxus*	Seafood	TGF	COI
DIF_070	Company 9	Processed product	*Merluccius hubbsi Marini*	*Merluccius gayi*	Seafood	TGF	COI
DIF_071	Company 10	Raw plant	*Humulus lupulus* L.	*Humulus lupulus* L.	Botanicals	DPQ	ITS
DIF_072	Company 10	Extraction waste 7% EtOH	*Humulus lupulus* L.	*Humulus lupulus* L.	Botanicals	DPQ/CTAB	ITS
DIF_073	Company 10	Extraction waste 50% EtOH	*Humulus lupulus* L.	*Humulus lupulus* L.	Botanicals	DPQ/CTAB	ITS
DIF_074	Company 10	Liquid phytoextract 7% EtOH	*Humulus lupulus* L.	*Humulus lupulus* L.	Botanicals	DPQ/CTAB	ITS
DIF_075	Company 10	Liquid phytoextract 50% EtOH	*Humulus lupulus* L.	*Humulus lupulus* L.	Botanicals	DPQ/CTAB	ITS
DIF_076	Company 11	Powder	*Malva sylvestris* L.	*Malva sylvestris* L.	Botanicals	DPQ	ITS
DIF_077	Company 11	Powder	*Carica papaya* L.	*Contamination*	Botanicals	DPQ	ITS
DIF_078	Company 11	Powder	*Valeriana officinalis* L.	*Valeriana officinalis* L.	Botanicals	DPQ	*psbA-trnH*
DIF_079	Company 11	Dry phytoextract	*Garcinia cambogia Desr.*	*Jurinea leptoloba*	Botanicals	DPQ/CTAB	ITS
DIF_080	Company 11	Liquid idroalcolic phytoextract	*Vaccinium macrocarpon Aiton*	*Leersia* spp.	Botanicals	DPQ/CTAB	rbcL
DIF_081	Company 11	Dry phytoextract CO2 supercritical	*Magnolia officinalis Rehder & E.H. Wilson*	*No DNA detected*	Botanicals	DPQ/CTAB	ITS
DIF_082	Company 12	Essential oil	*Citrus limon* L.	*No DNA detected*	Botanicals	DPQ/CTAB	ITS
DIF_083	Company 12	Essential oil	*Mentha x piperita* L.	*Abotilum indicum*	Botanicals	DPQ/CTAB	ITS
DIF_084	Company 12	Essential oil	*Citrus sinensis* L.	*Contamination*	Botanicals	DPQ/CTAB	ITS
DIF_085	Company 12	Liquid gliceric phytoextract	*Matricaria chamomilla* L.	*Sida acuta*	Botanicals	DPQ/CTAB	*matK*
DIF_086	Company 12	Liquid gliceric phytoextract	*Camellia sinensis Kuntze*	*No DNA detected*	Botanicals	DPQ/CTAB	ITS
DIF_087	Company 12	Liquid gliceric phytoextract	*Calendula officinalis* L.	*No DNA detected*	Botanicals	DPQ/CTAB	*psbA-trnH*
DIF_088	Company 12	Flour	*Oryza sativa* L.	*Cicer arietinum*	Botanicals	DPQ/CTAB	ITS
DIF_089	Company 12	Flour	*Manihot esculenta Crantz*	*Cicer arietinum*	Botanicals	DPQ/CTAB	ITS
DIF_090	Company 12	Powder	*Aloe vera* L.	*No DNA detected*	Botanicals	DPQ/CTAB	*psbA-trnH*
DIF_091	Company 13	Raw plant	*Cynara cardunculus* L.	*Cynara cardunculus* L.	Botanicals	DPQ	*psbA-trnH*
DIF_092	Company 13	Dry acqueous phytoextract	*Cynara cardunculus* L.	*Contamination*	Botanicals	DPQ/CTAB	*psbA-trnH*
DIF_093	Company 14	Food supplement	*Panax ginseng C.A. Meyer*	*Contamination*	Botanicals	DPQ/CTAB	*rbcL*
DIF_094	Company 14	Food supplement	*Monascus purpureus*	*Contamination*	Botanicals	DPQ/CTAB	ITS
DIF_095	Company 14	Raw plant	*Aloe vera* L.	*Contamination*	Botanicals	DPQ/CTAB	*psbA-trnH*
DIF_096	Company 15	Raw plant	*Syzygium aromaticum* (L.) *Merr & L.M. Perry*	*Contamination*	Botanicals	DPQ/CTAB	ITS
DIF_097	Company 15	Powder	*Rhus* spp.	*No DNA detected*	Botanicals	DPQ/CTAB	ITS
DIF_098	Company 15	Powder	*Citrus hystrix DC.*	*Moniliella suaveolens*	Botanicals	DPQ/CTAB	ITS
DIF_099	Company 16	Food supplement	*Phyllanthus niruri* L.	*No DNA detected*	Botanicals	DPQ/CTAB	ITS
DIF_100	Company 16	Food supplement	*Hibiscus sabdariffa* L.	*No DNA detected*	Botanicals	DPQ/CTAB	ITS
DIF_101	Company 16	Oil	*Serenoa repens Small*	*No DNA detected*	Botanicals	DPQ/CTAB	ITS
DIF_102	Company 17	Raw plant	*Cymbopogon citratus Stapf*	*Cymbopogon citratus Stapf*	Botanicals	DPQ	*psbA-trnH*
DIF_103	Company 17	Raw plant	*Glycyrrhiza glabra* L.	*Glycyrrhiza glabra* L.	Botanicals	DPQ	*matK* + ITS
DIF_104	Company 17	Raw plant	*Foeniculum vulgare Mill.*	*Foeniculum vulgare Mill.*	Botanicals	DPQ	*psbA-trnH*
DIF_105	Company 17	Raw plant	*Malva sylvestris* L.	*Malva sylvestris* L.	Botanicals	DPQ	*psbA-trnH*
DIF_106	Company 17	Raw plant	*Matricaria chamomilla* L.	*Matricaria chamomilla* L.	Botanicals	DPQ	*matK*
DIF_107	Company 17	Raw plant	*Matricaria chamomilla* L.	*Matricaria chamomilla* L.	Botanicals	DPQ	*matK*
DIF_108	Company 17	Raw plant	*Foeniculum vulgare Mill.*	*Foeniculum vulgare Mill.*	Botanicals	DPQ	*psbA-trnH*
DIF_109	Company 17	Raw plant	*Zingiber officinale Roscoe*	*Zingiber officinale Roscoe*	Botanicals	DPQ	ITS
DIF_110	Company 17	Raw plant	*Citrus limon* L.	*Contamination*	Botanicals	DPQ	ITS
DIF_111	Company 17	Raw plant	*Citrus limon* L.	*Citrus limon* L.	Botanicals	DPQ	ITS
DIF_112	Company 18	Raw plant	*Camellia sinensis Kuntze*	*Camellia sinensis var. sinensis*	Botanicals	DPQ	ITS
DIF_113	Company 19	Liquid phyotoextract 23% EtOH	*Althaea officinalis* L.	*Althaea officinalis* L.	Botanicals	DPQ/CTAB	*rbcL* mini-barcoding
DIF_114	Company 19	Dry phytoextract CO2 supercritical	*Serenoa repens Small*	*Contamination*	Botanicals	DPQ/CTAB	ITS
DIF_115	Company 19	Liquid gliceric phytoextract	*Althaea officinalis* L.	*No DNA detected*	Botanicals	DPQ/CTAB	*rbcL*
DIF_116	Company 19	Liquid gliceric phytoextract	*Althaea officinalis* L.	*No DNA detected*	Botanicals	DPQ/CTAB	*rbcL*
DIF_117	Company 19	Liquid phyotoextract 23% EtOH	*Althaea officinalis* L.	*Althaea officinalis* L.	Botanicals	DPQ/CTAB	*rbcL* mini-barcoding
DIF_118	Company 20	Dry phyotoextract 23% EtOH	*Vaccinium myrtillus* L.	*No DNA detected*	Botanicals	DPQ/CTAB	*rbcL*
DIF_119	Company 20	Dry phyotoextract 23% EtOH	*Vaccinium myrtillus* L.	*No DNA detected*	Botanicals	DPQ/CTAB	*rbcL*
DIF_120	Company 20	Dry phyotoextract	*Panax ginseng C.A. Meyer*	*Contamination*	Botanicals	DPQ/CTAB	*rbcL*
DIF_121	Company 20	Dry phytoextract	*Panax ginseng C.A. Meyer*	*No DNA detected*	Botanicals	DPQ/CTAB	*rbcL*
DIF_122	Company 20	Dry phytoextract	*Curcuma longa* L.	*No DNA detected*	Botanicals	DPQ/CTAB	ITS
DIF_123	Company 20	Powder	*Malva sylvestris* L.	*Contamination*	Botanicals	DPQ	*psbA-trnH*
DIF_124	Company 21	Raw plant	*Origanum vulgare* L.	*Origanum onites*	Botanicals	DPQ	ITS
DIF_125	Company 21	Processed product	*Prunus dulcis (Mill.) D.A.Webb*	*Prunus dulcis (Mill.) D.A.Webb*	Botanicals	DPQ	ITS
DIF_126	Company 22	Dry phytoextract	*Magnolia officinalis Rehder & E.H. Wilson*	*No DNA detected*	Botanicals	DPQ/CTAB	ITS
DIF_127	Company 22	Powder	*Plantago ovata Forssk.*	*Plantago* spp.	Botanicals	DPQ	*psbA-trnH*
DIF_128	Company 22	Powder	*Plantago ovata Forssk.*	*Plantago ovata*	Botanicals	DPQ	*psbA-trnH*
DIF_129	Company 22	Dry phytoextract	*Elaeis guineensis Jacq.*	*No DNA detected*	Botanicals	DPQ/CTAB	ITS
DIF_130	Company 22	Dry phytoextract	*Carum carvi* L.	*No DNA detected*	Botanicals	DPQ/CTAB	*matK*
DIF_131	Company 23	Raw plant	*Silybum marianum* (L.) *Gaertn.*	*Contamination*	Botanicals	DPQ	ITS
DIF_132	Company 23	Dry phytoextract	*Silybum marianum* (L.) *Gaertn.*	*No DNA detected*	Botanicals	DPQ/CTAB	ITS
DIF_133	Company 24	Powder	*Plantago ovata Forssk.*	*Plantago ovata Forssk.*	Botanicals	DPQ	*psbA-trnH*
DIF_134	Company 25	Raw plant	*Cymbopogon citratus Stapf*	*Cymbopogon citratus Stapf*	Botanicals	DPQ	*psbA-trnH*
DIF_135	Company 25	Raw plant	*Citrus limon* L.	*Citrus limon* L.	Botanicals	DPQ	ITS
DIF_136	Company 26	Powder	*Crocus sativus* L.	*Contamination*	Botanicals	DPQ	*matK* + ITS
DIF_137	Company 29	Extraction waste	*Zingiber officinale Roscoe*	*No DNA detected*	Botanicals	DPQ/CTAB	ITS
DIF_138	Company 29	Liquid acqueous phytoexctract	*Zingiber officinale Roscoe*	*Zingiber officinale Roscoe*	Botanicals	DPQ/CTAB	ITS
DIF_139	Company 29	Essential oil	*Zingiber officinale Roscoe*	*No DNA detected*	Botanicals	DPQ/CTAB	ITS
DIF_140	Company 30	Liquid phytoexctract	*Panax ginseng C.A. Meyer*	*No DNA detected*	Botanicals	DPQ/CTAB	rbcL
DIF_141	Company 15	Powder	*Cinnamomum verum J.Presl*	*Contamination*	Spice	DPQ	*matK* + *psbA-trnH*
DIF_142	Company 15	Raw plant	*Cinnamomum verum J.Presl*	*Cinnamomum* spp.	Spice	DPQ	*matK* + *psbA-trnH*
DIF_143	Company 15	Raw plant	*Piper borbonense C. DC.*	*Piper guineense*	Spice	DPQ	ITS
DIF_144	Company 15	Raw plant	*Vanilla planifolia Jacks. ex Andrews*	*Contamination*	Spice	DPQ	ITS
DIF_145	Company 15	Raw plant	*Vanilla planifolia Jacks. ex Andrews*	*No DNA detected*	Spice	DPQ/CTAB	ITS
DIF_146	Company 26	Powder	*Curcuma longa* L.	*Contamination*	Spice	DPQ	ITS
DIF_147	Company 26	Powder	*Curcuma longa* L.	*Curcuma* spp.	Spice	DPQ	ITS
DIF_148	Company 26	Raw plant	*Cinnamomum cassia J.Presl*	*Cinnamomum cassia J.Presl*	Spice	DPQ	*matK* + *psbA-trnH*
DIF_149	Company 26	Raw plant	*Cinnamomum verum J.Presl*	*Cinnamomum verum J.Presl*	Spice	DPQ	*matK* + *psbA-trnH*
DIF_150	Company 26	Powder	*Cinnamomum cassia J.Presl*	*Cinnamomum cassia J.Presl*	Spice	DPQ	*matK* + *psbA-trnH*
DIF_151	Company 26	Raw plant	*Cinnamomum tamala T.Nees & Eberm.*	*Cinnamomum tamala T.Nees & Eberm.*	Spice	DPQ	*matK* + *psbA-trnH*
DIF_152	Company 26	Powder	*Zingiber officinale Roscoe*	*Contamination*	Spice	DPQ	ITS
DIF_153	Company 11	Powder	*Curcuma longa* L.	*Curcuma longa* L.	Spice	DPQ	ITS
DIF_154	Company 26	Powder	*Crocus sativus* L.	*Crocus sativus* L.	Spice	DPQ	*matK* + ITS
DIF_155	Company 26	Raw plant	*Crocus sativus* L.	*Crocus sativus* L.	Spice	DPQ	*matK* + ITS
DIF_156	Company 26	Powder	*Capsicum* spp.	*Contamination*	Spice	DPQ	ITS
DIF_157	Company 26	Raw plant	*Piper nigrum* L.	*Contamination*	Spice	DPQ	ITS
DIF_158	Company 26	Powder	*Capsicum* spp.	*Capiscum* spp.	Spice	DPQ	ITS
DIF_159	Company 26	Raw plant	*Vanilla planifolia Jacks. ex Andrews*	*Contamination*	Spice	DPQ	rbcL
DIF_160	Company 26	Raw plant	*Crocus sativus* L.	*Crocus sativus* L.	Spice	DPQ	*matK* + ITS
DIF_161	Company 27	Dry phytoextract 30% EtOH	*Crocus sativus* L.	*Contamination*	Spice	DPQ/CTAB	*matK* + ITS
DIF_162	Company 15	Powder	*Capsicum* spp.	*Capsicum* spp.	Spice	DPQ	ITS
DIF_163	Company 15	Powder	*Myristica fragrans Houtt.*	*Cuminum cyminum*	Spice	DPQ	ITS
DIF_164	Company 15	Powder	*Curcuma longa* L.	*Curcuma longa* L.	Spice	DPQ	ITS
DIF_165	Company 28	Raw plant	*Origanum vulgare* L.	*Origanum vulgare* L.	Spice	DPQ	ITS
DIF_166	Company 29	Raw plant	*Zingiber officinale Roscoe*	*Zingiber officinale Roscoe*	Spice	DPQ	ITS
DIF_167	Company 31	Raw plant	*Piper nigrum* L.	*Piper nigrum* L.	Spice	DPQ	ITS
DIF_168	Company 31	Raw plant	*Piper nigrum* L.	*Piper nigrum* L.	Spice	DPQ	ITS
DIF_169	Company 31	Raw plant	*Allium cepa* L.	*Allium cepa* L.	Spice	DPQ	ITS
DIF_170	Company 31	Powder	*Allium cepa* L.	*Allium cepa* L.	Spice	DPQ	ITS
DIF_171	Company 31	Powder	*Capsicum* spp.	*Capsicum* spp.	Spice	DPQ	ITS
DIF_172	Company 31	Powder	*Capsicum* spp.	*Capsicum* spp.	Spice	DPQ	ITS
DIF_173	Company 31	Powder	*Curcuma longa* L.	*Curcuma* spp.	Spice	DPQ	ITS
DIF_174	Company 31	Powder	*Curcuma longa* L.	*Curcuma longa L.*	Spice	DPQ	ITS
DIF_175	Company 31	Powder	*Elettaria cardamomum* (L.) *Maton*	*Elettaria cardamomum* (L.) *Maton*	Spice	DPQ	ITS
DIF_176	Company 31	Powder	*Elettaria cardamomum* (L.) *Maton*	*Elettaria cardamomum* (L.) *Maton*	Spice	DPQ	ITS
DIF_177	Company 31	Powder	*Cinnamomum cassia J.Presl*	*No DNA detected*	Spice	DPQ	*matK* + *psbA-trnH*
DIF_178	Company 32	Powder	*Cinnamomum cassia J.Presl*	*No DNA detected*	Spice	DPQ	*matK* + *psbA-trnH*
DIF_179	Company 32	Raw plant	*Ocimum basilicum* L.	*Ocimum basilicum* L.	Spice	DPQ	ITS
DIF_180	Company 32	Powder	*Myristica* spp.	*Myristica fragrans*	Spice	DPQ	ITS
DIF_181	Company 32	Powder	*Capsicum annuum* L.	*Capsicum* spp.	Spice	DPQ	ITS
DIF_182	Company 32	Raw plant	*Piper nigrum* L.	*Piper nigrum* L.	Spice	DPQ	ITS
DIF_183	Company 33	Powder	*Fragaria* spp.	*Fragaria* spp.	Agrifood	DPQ	ITS
DIF_184	Company 34	Fresh product	*Tuber melanosporum vitt.*	*Tuber melanosporum vitt.*	Agrifood	DPQ	ITS
DIF_185	Company 34	Fresh product	*Tuber aestivum vitt.*	*Tuber aestivum vitt.*	Agrifood	DPQ	ITS
DIF_186	Company 34	Fresh product	*Tuber magnatum pico*	*Tuber magnatum pico*	Agrifood	DPQ	ITS
DIF_187	Company 34	Fresh product	*Tuber uncinatum chatin*	*Tuber uncinatum chatin*	Agrifood	DPQ	ITS
DIF_188	Company 34	Fresh product	*Tuber albidum pico*	*Tuber albidum pico*	Agrifood	DPQ	ITS
DIF_189	Company 34	Fresh product	*Tuber aestivum vitt.*	*Tuber aestivum vitt.*	Agrifood	DPQ	ITS
DIF_190	Company 34	Fresh product	*Tuber albidum pico*	*Tuber albidum pico*	Agrifood	DPQ	ITS
DIF_191	Company 34	Fresh product	*Tuber uncinatum chatin*	*Tuber uncinatum/aestivum*	Agrifood	DPQ	ITS
DIF_192	Company 34	Fresh product	*Tuber magnatum Pico.*	*Tuber magnatum Pico.*	Agrifood	DPQ	ITS
DIF_193	Company 34	Fresh product	*Tuber brumale Vitt.*	*Tuber melanosporum*	Agrifood	DPQ	ITS
DIF_194	Company 34	Fresh product	*Tuber mesentericum Vitt.*	*Tuber mesentericum*	Agrifood	DPQ	ITS
DIF_195	Company 34	Fresh product	*Tuber brumale Vitt.*	*Tuber brumale Vitt.*	Agrifood	DPQ	ITS
DIF_196	Company 15	Fresh product	*Theobroma cacao* L.	*Theobroma cacao* L.	Agrifood	DPQ	ITS
DIF_197	Company 11	Powder	*Carica papaya* L.	*Prunus* spp.	Agrifood	DPQ	ITS
DIF_198	Company 35	Flour	*Oryza sativa* L.	*Cicer arietinum*	Agrifood	DPQ	ITS
DIF_199	Company 35	Flour	*Oryza sativa* L.	*Cicer arietinum*	Agrifood	DPQ	ITS
DIF_200	Company 24	Food supplement liophilized	*Lactobacillus paracasei*	*Lactobacillus paracasei*	Probiotics	QAQ	16S rRNA
DIF_201	Company 36	Food supplement liophilized	*Lactobacillus gasseri*	*Lactobacillus gasseri*	Probiotics	QAQ	16S rRNA
DIF_202	Company 36	Food supplement liophilized	*Lactobacillus gasseri*	*Lactobacillus gasseri*	Probiotics	QAQ	16S rRNA
DIF_203	Company 36	Food supplement liophilized	*Lactobacillus reuteri*	*Lactobacillus reuteri*	Probiotics	QAQ	16S rRNA
DIF_204	Company 36	Food supplement liophilized	*Lactobacillus paracasei*	*Lactobacillus paracasei*	Probiotics	QAQ	16S rRNA
DIF_205	Company 37	Food supplement liophilized	*Lactobacillus paracasei*	*Lactobacillus paracasei*	Probiotics	QAQ	16S rRNA
DIF_206	Company 37	Food supplement liophilized	*Lactobacillus acidophilus*	*Lactobacillus acidophilus*	Probiotics	QAQ	16S rRNA
DIF_207	Company 37	Food supplement liophilized	*Lactobacillus acidophilus*	*Lactobacillus acidophilus*	Probiotics	QAQ	16S rRNA
DIF_208	Company 37	Food supplement liophilized	*Bifidobacterium lactis*	*Bifidobacterium lactis*	Probiotics	QAQ	16S rRNA
DIF_209	Company 37	Food supplement liophilized	*Bifidobacterium lactis*	*Bifidobacterium lactis*	Probiotics	QAQ	16S rRNA
DIF_210	Company 38	Food supplement liophilized	*Bifidobacterium bifidum*	*Contamination*	Probiotics	QAQ	16S rRNA
DIF_211	Company 38	Food supplement liophilized	*Bifidobacterium bifidum*	*Bifidobacterium bifidum*	Probiotics	QAQ	16S rRNA
DIF_212	Company 38	Food supplement liophilized	*Bifidobacterium bifidum*	*Bifidobacterium bifidum*	Probiotics	QAQ	16S rRNA

**Table A2:** In the table are indicated the PCR program for all the couples of primers used in the study.

	Cox1_Ward_FishF1 Cox1_Ward_FishR1	Cox1_Ward_FishF2 Cox1_Ward_FishR2	LCO 1490 HCO 2198	16sar-L 16sbr_H	GLUDG C61221H	Tuna_CR_F Tuna_CR_R	Tuna_CR_F Tuna_minibar_R2	Sco5S_F Sco5S_R	Katw_F Katw_R	Dlab_F Dlab_R	rbcL_1F rbcL724R	rbcL 1 rbcL B	matK_3F_KIM matK_1R_KIM	psbA trnH	ITS-p5 ITS-u4	ITS3_KYO2 ITS-4	P0 P6
**Initial step**	94 °C for 3′	94 °C for 3′	94 °C for 3′	94 °C for 3′	94 °C for 3′	94 °C for 3′	94 °C for 3′	94 °C for 3′	94 °C for 3′	94 °C for 3′	94 °C for 3′	94 °C for 3′	94 °C for 3′	94 °C for 3′	94 °C for 3′	94 °C for 3′	94 °C for 3′
**N. of cycles**	35	35	35	40	35	35	35	35	35	30	35	35	35	35	35	35	33
**Denaturation**	94 °C for 25″	94 °C for 25″	94 °C for 1′	94 °C for 25″	94 °C for 1′	94 °C for 30″	94 °C for 30″	94 °C for 45″	94 °C for 30″	94 °C for 30″	94 °C for 45″	94 °C for 45″	94 °C for 45″	94 °C for 45″	94 °C for 45″	94 °C for 45″	94 °C for 20″
**Annealing**	55 °C for 25″	55 °C for 25″	47 °C for 90″	57 °C for 15″	52 °C for 1′	58 °C for 40″	52 °C for 40″	48 °C for 45″	54 °C for 40″	59 °C for 40″	50 °C for 30″	50 °C for 30″	53 °C for 30″	53 °C for 30″	55 °C for 30″	55 °C for 30″	54 °C for 30″
**Extention**	72 °C for 1′	72 °C for 1′	72 °C for 25″	72 °C for 20″	72 °C for 3′	72 °C for 1′	72 °C for 1′	72 °C for 1′	72 °C for 1′	72 °C for 1′	72 °C for 1′	72 °C for 1′	72 °C for 1′	72 °C for 1′	72 °C for 1′	72 °C for 1′	72 °C for 45″
**Final elongation**	72 °C for 10′	72 °C for 10′	72 °C for 7′	72 °C for 10′	70 °C for 5′	72 °C for 10′	72 °C for 10′	72 °C for 7′	72 °C for 10′	72 °C for 10′	72 °C for 7′	72 °C for 7′	72 °C for 7′	72 °C for 7′	72 °C for 7′	72 °C for 7′	72 °C for 5′
